# Supplementary material for: Temporal transcription factors determine circuit membership by permanently altering motor neuron-to-muscle synaptic partnerships
Source: eLife. 2020 May 11;9:e56898. doi: 10.7554/eLife.56898 (PMC7242025; doi:10.7554/eLife.56898)
Supplement: Figure 1—source data 1. [file elife-56898-fig1-data1.docx]

Source Data for Figure 1K-P

|  | Genotype | Number of values | Mean  (branch number) | Std. Deviation | Std. Error of Mean | p value |
| --- | --- | --- | --- | --- | --- | --- |
| muscle 12-1s | UAS-Hb/+ | 22 | 1.273 | 0.4558 | 0.09719 | NA |
| Figure K | NB3-1/+ | 30 | 1.233 | 0.4302 | 0.07854 | 0.9846 |
|  | NB3-1>Hb | 39 | 0.02564 | 0.1601 | 0.02564 | <0.0001* |
|  |  |  |  |  |  |  |
| muscle 12-1b | UAS-Hb/+ | 21 | 1.857 | 0.5732 | 0.1251 | NA |
| Figure L | NB3-1/+ | 30 | 1.933 | 0.4498 | 0.08212 | 0.9403 |
|  | NB3-1>Hb | 38 | 1.842 | 0.6789 | 0.1101 | 0.9996 |
|  |  |  |  |  |  |  |
| Muscle 30-1b | UAS-Hb/+ | 22 | 1.091 | 0.2942 | 0.06273 | NA |
| Figure M | NB3-1/+ | 29 | 1.310 | 0.4708 | 0.08743 | 0.1331 |
|  | NB3-1>Hb | 39 | 2.923 | 0.9565 | 0.1532 | <0.0001* |
|  |  |  |  |  |  |  |
| muscle 14-1b | UAS-Hb/+ | 22 | 2.227 | 0.5284 | 0.1127 | NA |
| Figure N | NB3-1/+ | 29 | 1.862 | 0.5158 | 0.9577 | 0.0508 |
|  | NB3-1>Hb | 39 | 4.744 | 1.141 | 0.1826 | <0.0001* |
|  |  |  |  |  |  |  |
| muscle 6/7-1b | UAS-Hb/+ | 21 | 4.286 | 1.617 | 0.3528 | NA |
| Figure O | NB3-1/+ | 30 | 4.700 | 1.653 | 0.3000 | 0.7521 |
|  | NB3-1>Hb | 38 | 1.974 | 1.241 | 0.2013 | <0.0001* |
|  |  |  |  |  |  |  |
| muscle 13-1b | UAS-Hb/+ | 21 | 1.619 | 0.6690 | 0.1460 | NA |
| Figure P | NB3-1/+ | 29 | 1.241 | 0.4355 | 0.08087 | 0.0876 |
|  | NB3-1>Hb | 39 | 1.538 | 0.6426 | 0.1029 | 0.9573 |

*Brown-Forsythe and Welch ANOVA (for un-equal Std. Deviation) with Dunnett correction for multiple comparison
