# Supplementary material for: Temporal transcription factors determine circuit membership by permanently altering motor neuron-to-muscle synaptic partnerships
Source: eLife. 2020 May 11;9:e56898. doi: 10.7554/eLife.56898 (PMC7242025; doi:10.7554/eLife.56898)
Supplement: Figure 1—figure supplement 2—source data 1. [file elife-56898-fig1-figsupp2-data1.docx]

Source Data for Figure 1—figure supplement 2F

| Genotype | Marker | Number of values | Mean (number of cells) | Std. Deviation | Std. Error of Mean | p value |
| --- | --- | --- | --- | --- | --- | --- |
| Control | Kr+ Zfh2- | 31 | 2.000 | 0.000 | 0.000 | NA |
| NB3-1>Hb | Kr+ Zfh2- | 60 | 5.633 | 1.461 | 0.1886 | <0.0001* |
|  |  |  |  |  |  |  |
| Control | Kr+ Zfh2+ | 31 | 1.000 | 0.000 | 0.000 | NA |
| NB3-1>Hb | Kr+ Zfh2+ | 90 | 0.1167 | 0.3237 | 0.04179 | <0.0001* |
|  |  |  |  |  |  |  |
| Control | Hb- Cut+ | 31 | 1.000 | 0.000 | 0.000 | NA |
| NB3-1>Hb | Hb- Cut+ | 60 | 0.111 | 0.3160 | 0.03331 | <0.0001* |

* t test, with Welch’s correction (for un-equal Std. Deviation)
