# Supplementary material for: Temporal transcription factors determine circuit membership by permanently altering motor neuron-to-muscle synaptic partnerships
Source: eLife. 2020 May 11;9:e56898. doi: 10.7554/eLife.56898 (PMC7242025; doi:10.7554/eLife.56898)
Supplement: Figure 3—source data 1. [file elife-56898-fig3-data1.docx]

Source Data for Figure 3D-F

| For Muscle 14 | Genotype | Number of values | Mean | Std. Deviation | Std. Error of Mean | p value |
| --- | --- | --- | --- | --- | --- | --- |
| EPSP (mV) | NB3-1/+ | 8 | 24.38 | 3.187 | 1.127 | NA |
| Figure H | NB3-1>Hb | 8 | 22.72 | 5.701 | 2.016 | 0.4863 |
|  |  |  |  |  |  |  |
| mEPSP amp. (mV) | NB3-1/+ | 8 | 1.116 | 0.2278 | 0.08054 | NA |
| Figure I | NB3-1>Hb | 8 | 0.7287 | 0.06789 | 0.02400 | 0.0016** |
|  |  |  |  |  |  |  |
| Quantal Content | NB3-1/+ | 8 | 22.59 | 5.405 | 1.911 | NA |
| Figure J | NB3-1>Hb | 8 | 31.32 | 8.294 | 2.932 | 0.0281** |

Source Data for Figure 3I-K

| For Muscle 6 | Genotype | Number of values | Mean | Std. Deviation | Std. Error of Mean | p value |
| --- | --- | --- | --- | --- | --- | --- |
| EPSP (mV) | NB3-1/+ | 17 | 36.45 | 4.690 | 1.138 | NA |
| Figure C | NB3-1>Hb | 21 | 22.94 | 10.37 | 2.262 | <0.0001** |
|  |  |  |  |  |  |  |
| mEPSP amp. (mV) | NB3-1/+ | 16 | 1.083 | 0.1875 | 0.04689 | NA |
| Figure D | NB3-1>Hb | 21 | 1.207 | 0.2242 | 0.04892 | 0.0743 |
|  |  |  |  |  |  |  |
| Quantal Content | NB3-1/+ | 16 | 35.15 | 8.8154 | 2.038 | NA |
| Figure E | NB3-1>Hb | 21 | 19.36 | 8.828 | 1.926 | <0.0001* |

* Unpaired t test

**Unpaired t-test, with Welch’s correction (for un-equal Std. Deviation)
