# Supplementary material for: Temporal transcription factors determine circuit membership by permanently altering motor neuron-to-muscle synaptic partnerships
Source: eLife. 2020 May 11;9:e56898. doi: 10.7554/eLife.56898 (PMC7242025; doi:10.7554/eLife.56898)
Supplement: Figure 4—source data 1. [file elife-56898-fig4-data1.docx]

Source Data for Figure 4K-N

|  | Genotype | Number of values | Mean  (1b branch number) | Std. Deviation | Std. Error of Mean | p value |
| --- | --- | --- | --- | --- | --- | --- |
| muscle 9/10 | UAS-Cas/+ | 59 | 2.153 | 0.3626 | 0.04721 | NA |
| Figure K | NB7-1>Cas 3 Eve | 46 | 2.130 | 0.4993 | 0.07361 | 0.9920 |
|  | NB7-1>Cas 4 Eve | 40 | 1.950 | 0.5038 | 0.07966 | 0.0930 |
|  |  |  |  |  |  |  |
| muscle 2 | UAS-Cas/+ | 30 | 2.300 | 0.4661 | 0.08510 | NA |
| Figure L | NB7-1>Cas 3 Eve | 23 | 1.957 | 0.4747 | 0.09897 | 0.0337 |
|  | NB7-1>Cas 4 Eve | 20 | 1.950 | 0.5104 | 0.1141 | 0.0542 |
|  |  |  |  |  |  |  |
| muscle 3 | UAS-Cas/+ | 30 | 1.800 | 0.4068 | 0.07428 | NA |
| Figure M | NB7-1>Cas 3 Eve | 23 | 0.3478 | 0.6473 | 0.1350 | <0.0001* |
|  | NB7-1>Cas 4 Eve | 20 | 0.9000 | 0.6407 | 0.1433 | <0.0001* |
|  |  |  |  |  |  |  |
| muscle 4 | UAS-Cas/+ | 30 | 2.133 | 0.5074 | 0.09264 | NA |
| Figure N | NB7-1>Cas 3 Eve | 23 | 0.7826 | 0.7952 | 0.1658 | <0.0001* |
|  | NB7-1>Cas 4 Eve | 20 | 1.350 | 0.7452 | 0.1666 | 0.0008* |

*Brown-Forsythe and Welch ANOVA (for un-equal Std. Deviation) with Dunnett correction for multiple comparison
