# Supplementary material for: Temporal transcription factors determine circuit membership by permanently altering motor neuron-to-muscle synaptic partnerships
Source: eLife. 2020 May 11;9:e56898. doi: 10.7554/eLife.56898 (PMC7242025; doi:10.7554/eLife.56898)
Supplement: Figure 5—source data 1. [file elife-56898-fig5-data1.docx]

Source Data for Figure 5M-P

|  | Genotype | Number of values | Mean  (1b branch number) | Std. Deviation | Std. Error of Mean | p value |
| --- | --- | --- | --- | --- | --- | --- |
| muscle 9/10 | UAS-Pdm/+ | 33 | 1.909 | 0.5222 | 0.09091 | NA |
| Figure M | NB7-1>Pdm 4 Eve | 45 | 1.756 | 0.5290 | 0.07885 | 0.4964 |
|  | NB7-1>Pdm 5 Eve | 16 | 2.083 | 0.4082 | 0.08333 | 0.5398 |
|  |  |  |  |  |  |  |
| muscle 2 | UAS-Pdm/+ | 26 | 2.308 | 0.6177 | 0.1211 | NA |
| Figure N | NB7-1>Pdm 4 Eve | 23 | 1.174 | 0.6503 | 0.1356 | <0.0001* |
|  | NB7-1>Pdm 5 Eve | 8 | 1.500 | 0.9258 | 0.3273 | 0.1230 |
|  |  |  |  |  |  |  |
| muscle 3 | UAS-Pdm/+ | 28 | 1.571 | 0.5040 | 0.09524 | NA |
| Figure O | NB7-1>Pdm 4 Eve | 22 | 1.955 | 0.8985 | 0.1916 | 0.2249 |
|  | NB7-1>Pdm 5 Eve | 8 | 2.500 | 1.309 | 0.4629 | 0.2168 |
|  |  |  |  |  |  |  |
| muscle 4 | UAS-Pdm/+ | 28 | 2.143 | 0.6506 | 0.1230 | NA |
| Figure P | NB7-1>Pdm 4 Eve | 23 | 2.130 | 0.8149 | 0.1699 | 0.9999 |
|  | NB7-1>Pdm 5 Eve | 8 | 6.500 | 2.138 | 0.7559 | 0.0021** |

*Ordinary one-way ANOVA with Dunnett correction for multiple comparison

**Brown-Forsythe and Welch ANOVA (for un-equal Std. Deviation) with Dunnett correction for multiple comparison
